# Vascular contributions to pattern analysis: Comparing gradient and spin echo fMRI at 3T

**DOI:** 10.1016/j.neuroimage.2010.03.061

**Published:** 2011-05-15

**Authors:** Russell Thompson, Marta Correia, Rhodri Cusack

**Affiliations:** MRC Cognition and Brain Sciences Unit, 15 Chaucer Road, Cambridge, CB2 7EF, UK

**Keywords:** fMRI, Multivariate pattern analysis, Classification, Decoding, Spin echo

## Abstract

Multivariate pattern analysis is often assumed to rely on signals that directly reflect differences in the distribution of particular neural populations. The source of the signal used in these analyses remains unclear however, and an alternative model suggests that signal from larger draining veins may play a significant role. The current study was designed to investigate the vascular contribution to pattern analyses at 3T by comparing the results obtained from gradient and spin echo data. Classification analyses were carried out comparing line orientations in V1, tone frequencies in A1, and responses from different fingers in M1. In all cases, classification accuracy in the spin echo data was not significantly different from chance. In contrast, classification accuracies in the gradient echo data were significantly above chance, and significantly higher than the accuracies observed for the spin echo data. These results suggest that at the field strength and spatial resolution used for the majority of fMRI studies, a considerable proportion of the signal used by pattern analysis originates in the vasculature.

## Introduction

Pattern recognition techniques and similar multivariate methods have provided a powerful new way of examining fMRI data, and over recent years they have been applied in a range of experimental contexts. One application that has generated particular interest is the use of pattern analysis to discriminate responses to individual stimuli or cognitive events that produce spatially overlapping activations and are therefore not easily identified by univarite analyses. This method is based on the idea that small differences in the signals from individual voxels, while they may not produce statistically significant results when studied in isolation, do contain information about stimulus conditions, and combining the information from multiple voxels can allow a separation of the responses associated with those conditions (Haxby et al., 2001; Boynton, 2005; Haynes and Rees, 2006; Norman et al, 2006). While the technique has been used with some success however, the source of the subtle biases present in individual voxels remains unclear, and there has been relatively little investigation into the nature of the distributed activity patterns that form the basis of the technique.

Perhaps the most widely accepted explanation is that weak differential signals within individual voxels reflect differences in the spatial distribution of the underlying neural populations (Boynton, 2005; Kamitani and Tong, 2005; Haynes and Rees, 2006). Individual voxels provide an idiosyncratic or biased sample of these populations, with each voxel containing slightly different proportions of cells that are maximally sensitive to any given stimulus. Implicit within the idea of biased sampling is the assumption that the signal measured at each voxel represents a (more or less) direct average of the activity across its constituent neural populations. In reality, the situation is likely to be considerably more complex, as gradient echo (GE) sequences of the type typically used for fMRI provide a signal that originates from a mixture of sources (Ogawa et al, 1998). At a field strength of 3T, in addition to intra- and extra-vascular signals originating from the capillary bed and surrounding gray matter, a substantial proportion of the signal originates from in and around larger draining veins. This vascular signal limits the spatial specificity of gradient echo data, and several studies have demonstrated that the T2* weighted BOLD signals provided by GE sequences extend beyond the focus of neural activity (Ugurbil et al, 2003), with an estimated point spread function of 2–4 mm (Engel et al, 1997; Parkes et al, 2005; Shmuel et al, 2007a). This type of blurred vascular filtering should pose a problem if pattern analyses rely solely on sampling high spatial frequency information about different neural populations, as it reduces the correlation between the signal measured at the level of individual voxels and the activity of different neural populations within those voxels*.* There is also evidence that the presence of larger veins could lead to the BOLD signal being referred from distant sites of neural activity (Olman et al, 2007; see also Turner, 2002), also potentially reducing the specificity with which the signal from an individual voxel reflects its unique neural constituents.

An alternative model (Kamitani and Tong, 2005; Kriegeskorte et al, 2010; Gardner, 2010) suggests that rather than posing a problem for pattern analysis, the vascular signal may itself contain a considerable amount of information. According to the “complex spatio-temporal filter” model proposed by Kriegeskorte et al. (2010), the biases visible in individual voxels represent idiosyncrasies in the vasculature within those voxels and in the way in which the vasculature is coupled to different neural populations: in response to a particular stimulus event, characteristic patterns of neural response produce, in turn, a complex vascular signal due to differences in the way particular vessels drain certain populations. In this respect the model suggests multiple levels of biased sampling, firstly through the way in which the vasculature samples different neural populations, and secondly through the way the vascular pattern is itself sampled by the imaging sequence. Another possibility (Gardner, 2010) is that the structure of the vasculature may actually mirror the functional organisation observed in the gray matter, and common drainage of functional units with similar response properties may provide a vascular signal containing stimulus specific information.

There have been relatively few studies directly examining these hypotheses, but recent findings demonstrating that information about stimulus orientation (Gardner et al., 2006) and ocular dominance (Shmuel et al., 2010) can be decoded from voxels in the region of large blood vessels provide support for the idea of a macro-vascular contribution to pattern analysis. Simulations also suggest that this model would predict less sensitivity to spatial smoothing than a simple biased sampling model (Kriegeskorte et al., 2010), providing some consistency with recent results showing that downsampling (Gardner et al., 2006) or spatially smoothing (Op de Beeck et al., 2008; Op de Beeck, 2010) data are not necessarily harmful to classification.

In contrast to GE acquisition sequences, methods such as the Hahn Spin Echo have been shown to have higher spatial specificity with respect to the sites of neural activity (Thulborn et al., 1997; Goense and Logothetis, 2006; Yacoub et al., 2007). While spin echo data generally have a lower contrast-to-noise ratio than gradient echo data, the signal lost in spin echo sequences is specific to the extra-vascular signal around larger draining veins, with some further reduction in the intravascular signal from larger draining veins at higher magnetic field strengths and longer echo times (Lee et al., 1999; Duong et al., 2003; Jochimsen et al., 2004). The crucial question for pattern analytic studies therefore becomes whether this macro-vascular signal provides any useful information. On one hand, a non-specific signal from larger draining veins has the potential to blur activation patterns both within and across voxels, a situation that should reduce the amount of information available about the spatial distribution of neural populations. In contrast, if pattern analysis capitalises on complex patterns of neuro-vascular coupling or if the vasculature reflects the functional organisation within gray matter, gradient echo sequences may be optimal, as the signal originating from larger draining veins would be the primary factor contributing to differential activation patterns, especially at lower field strengths.

The study reported here was designed to investigate these issues further, focussing in particular on the contribution of larger vessels and the rate of spatial sampling. Participants were scanned using both a gradient echo (GE) and a spin echo (SE) EPI sequence at two different spatial resolutions while performing a task that allowed us to classify the activity patterns associated with different line orientations, tone frequencies, and responses from different fingers. If patterns of activity depend on differential distribution of neural populations, it would be predicted that sampling this information at a higher resolution and increasing the spatial specificity of the signal by using a spin echo sequence should improve classification accuracy. In contrast, the models proposed by Kriegeskorte et al. (2010) and Gardner (2010) would predict that classification accuracy should be reduced using the spin echo sequence due to the reduced vascular signal.

## Materials and methods

### Participants

The task was completed by 14 participants (five male; mean age 24 years 7 months, range 19–36 years). All participants were right handed, had normal or corrected to normal vision, and had no history of neurological or psychiatric illness. All participants were screened for MR contraindications and gave informed written consent prior to the start of the experiment. Participants received a small payment for taking part in the experiment. Three participants were excluded from further analysis due to excessive head movement.

### Task design and procedure

**Fig. 1:**
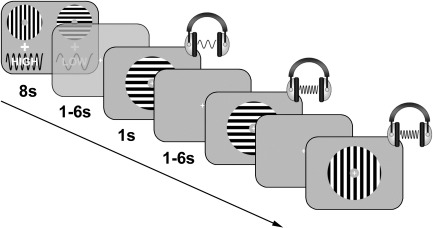
Schematic representation of the task. In each block of trials, participants associated one of two oriented gratings with one of two sine wave tones. On each experimental trial, participants were presented with a single grating/tone pair and asked to indicate whether the pair was correct or incorrect according to the current mapping.

Participants completed a paired associates task using two different oriented gratings (horizontal and vertical) and two different sine wave tones (low and high). Each block of trials began with a cue indicating which grating would be paired with which tone in the following block. Two pairings of orientation and tone were possible: vertical gratings could be paired with high tones and horizontal gratings with low tones (mapping 1) or vertical gratings could be paired with low tones and horizontal gratings with high tones (mapping 2). Cues were presented visually using a symbolic representation of the tones (Fig. 1) and were displayed for 8 s. After a variable interval of 1–6 s, participants were then presented with a series of 16 experimental trials. Each trial consisted of the simultaneous presentation of a single grating with a single tone, and the participants' task was to indicate whether the pairing was correct or incorrect according to the current mapping. Correct pairings were indicated by pressing a button underneath the index finger, and incorrect pairs by pressing a button under the middle finger. The task thus contained three separate dimensions on which classification analyses could be performed: orientation (horizontal vs. vertical), tone (high vs. low), and finger (index vs. middle).

Gratings used a full contrast square waveform with a frequency of approximately two cycles per degree. They were presented on a gray background (RGB 192, 192, 192) in an annular configuration with a diameter of approximately 8° of visual angle. The central aperture had a diameter of approximately 1.5° and contained a small white fixation cross in the centre. All visual stimuli were back-projected onto a screen located behind the bore of the magnet and viewed through a mirror mounted to the head coil. Auditory stimuli were presented via headphones, and the volume was adjusted for each participant at the beginning of the experiment so that both tones could be heard clearly above the noise of the scanner. High tones had a frequency of 1100 Hz, and low tones had a frequency of 220 Hz. All stimuli were presented for 1 s and followed by a variable interval of 1–6 s during which the fixation cross remained visible. Inter-stimulus intervals were taken from an exponential distribution in order to allow more efficient estimation of both transient responses to experimental stimuli and sustained responses across the course of each block (Chawla et al., 1999; Visscher et al., 2003).

Each block of trials contained four repetitions of each of the four possible orientation/tone pairings presented in pseudorandom order. After presentation of the final trial in each block there was a variable period of fixation (4–10 s) before the next block of trials began. Blocks of trials were grouped together in runs. Each run contained two blocks, one block using mapping 1 (vertical gratings paired with high tones and horizontal gratings paired with low tones) and one using mapping 2 (vertical/low and horizontal/high). The order in which blocks were presented was randomised within each run. Each run was followed by a variable interval of 12–18 s, and participants completed six runs in each scanning session. Participants completed four scanning sessions in total, each using a different acquisition sequence (see “MRI acquisition parameters” below). The order in which sessions were acquired was counterbalanced across participants.

### MRI acquisition parameters

Data were collected using a 3T Siemens Tim Trio scanner using a 12-channel transmit/receive birdcage head coil. Functional data were collected using four different EPI pulse sequences: standard resolution gradient echo (GE), high resolution GE, standard resolution spin echo (SE), and high resolution SE. Standard resolution sequences had a total field of view of 192 mm, with an in-plane resolution of 3 × 3 mm and a slice thickness of 3 mm plus 0.75 mm inter-slice interval. High resolution sequences had a total field of view of 192 mm, with an in-plane resolution of 2 × 2 mm and a slice thickness of 2 mm plus a 0.5 mm inter-slice interval. Both GE sequences used a flip angle of 82° and a TE of 30 ms, while the SE sequences used a flip angle of 90° and a TE of 72 ms. TE values were chosen give optimal BOLD contrast, approximating the T2* of gray matter in the GE sequence, and the T2 of gray matter in the SE sequence.

All functional scans had a TR of 2.49 s, allowing for a total acquisition of 40 slices in the standard resolution GE sequence, 40 slices in the high resolution GE sequence, 32 slices in the standard resolution SE sequence, and 23 slices in the high resolution SE sequence. An oblique axial orientation was used for all functional acquisitions, with the precise geometry being adjusted for each participant individually in order to maximise coverage of the primary visual, primary auditory, and primary motor cortices. The smaller volume of acquisition in the high resolution SE sequence meant that coverage of primary motor cortex was limited however, and no results are reported from the high resolution SE sequence in this region. Within each participant, the same angle of acquisition was used for all four functional scans.

In addition to the functional data, a high resolution (1 mm isotropic) T1 weighted structural image was collected using an MPRAGE sequence (TR = 2.25 s, TE = 2.98 ms, flip angle = 9°, matrix size  = 240 × 256 × 160).

### Data analysis

Data were analysed using SPM5 (Welcome Department of Cognitive Neurology, London, UK), together with the MarsBar toolbox (Brett et al., 2002) and custom Matlab functions. The first volumes from each scanning session were co-registered to the mean image from the standard resolution gradient echo scan, and the transformations derived from this step were applied to the remaining volumes within each scan. All volumes within each scanning session were then registered to the first volume from the relevant scan, and finally a slice time correction procedure was applied. In order to avoid T1 equilibration effects, the first eight volumes of each functional scan were discarded from further analysis. All functional data were also high pass filtered using a cut-off period of 120 s. Each participant's structural image was co-registered with the mean image from standard resolution gradient echo scan, segmented into gray matter, white matter and CSF partitions, and then normalised into the space defined by the MNI 152 template.

For each participant, a separate general linear model was fit to data from each of the scanning sessions. Within each session, each run of two blocks was modelled using 13 predictor variables. Experimental trials were modelled using eight predictor variables, with one predictor representing each of the eight possible combinations of orientation, tone and finger. Events were defined by which finger was actually pressed rather than according to which finger represented the correct response. An additional variable was used to model all trials where no response was made. One predictor variable was also included for each of the two possible cue stimuli, and one predictor was included to model the entire duration of each of the two possible block types (mapping 1 or mapping 2). All predictor variables were created by convolving a timing function indicating the onset and duration of each event type with a canonical haemodynamic response function. Parameters obtained from the spatial realignment procedure were also included as covariates in order to model head movements.

The parameter estimates obtained from each of these models were used as the input to a series of pattern classification analyses focussed on regions of interest in left and right primary visual cortex (V1), left and right primary auditory cortex (A1), and left primary motor cortex (M1; see “Regions of interest,” below). The parameter estimates for each of the eight experimental trial types in each of the six runs were obtained for all voxels falling within each of the regions of interest. Three separate classification analyses were then carried out on the data from each region, comparing values of orientation (vertical vs. horizontal), tone (low vs. high) and finger (index vs. middle). All classifications used a linear discriminant analysis with a shrinkage procedure to estimate the voxelwise covariance matrix (Ledoit and Wolf, 2004). A cross-validation test-train procedure was used for each classification, with the classifier trained on data from four runs and tested on data from two runs. Repeating this procedure for each possible combination of test and train sets gave a total of 15 iterations. Each run contained 4 exemplars of each stimulus value, so in each iteration the classifier was trained on 32 exemplars and tested on 16 exemplars. Each exemplar represented the response across an average of four stimulus events. The overall accuracy for each classification was found by averaging the accuracy scores from each test-train iteration. Finally, accuracy values for each participant were combined into a series of group analyses.

### Regions of interest

Coordinates for the V1 and A1 regions were obtained from the Jerne Volumes of Interest database (http://neuro.imm.dtu.dk/services/jerne/ninf/voi.html; Nielsen and Hansen, 2002; based on the BrainMap database, Fox et al., 1994). All coordinates listed under the terms “primary visual area,” “primary visual cortex” and “primary visual” were averaged together (taking the absolute value of the *X* coordinates), and the result was mirrored across hemispheres to give ± 10, −89, 1 as the centre points for the V1 regions. A similar procedure using the search terms “auditory cortex,” “gyrus heschl,” and “auditory” gave coordinates of ± 51, −19, 9 for the A1 regions.

In order to define M1 regions, univariate analyses were carried out based on the same general linear model described above but using normalised, smoothed (8 mm FWHM Gaussian kernel) data. A contrast was carried out to identify voxels that were significantly more active during experimental trials than during the fixation baseline (thresholded at *p* < 0.001, uncorrected for multiple comparisons). This was then masked to include only the voxels that fell within Brodmann Area 4 (defined using the template image provided with MRIcroN; http://www.sph.sc.edu/comd/rorden/mricron/), and the centre of mass of the remaining voxels was calculated. These points were averaged across subjects and sessions to give a central coordinate of −39, −22, 57.

All regions were defined by including the voxels that fell within a 10 mm radius of the centre coordinates. Regions were originally defined in MNI space, and the spatial transformations obtained from normalising each participant's structural image were then used to un-normalise these coordinates into the space of each individual participant's functional data. Each region was also masked to exclude all voxels that fell outside the brain or which had a probability of less than 0.1 of belonging to the gray matter partition.

## Results

The behavioural results showed that participants were able to perform the task with a high degree of accuracy and made correct responses on 91.2% of trials. Response rates, accuracy rates, and reaction times are presented in more detail in Table 1.

### fMRI pattern classifications results

**Fig. 2:**
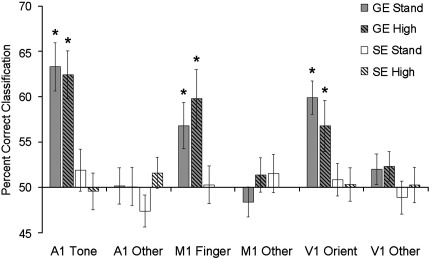
Mean classification accuracy rates for represented and non-represented stimulus dimensions in each region of interest for each type of acquisition sequence. Represented stimulus dimensions (i.e., tones in A1, fingers in M1, and orientations in V1) were discriminated with above chance accuracy in the gradient echo data but not in the spin echo data. * = 1 sample *t*-test against chance (50%), *p* < 0.05. Error bars represent 1 standard error of the mean (calculated across participants).

Fig. 2 shows the results of the classification analyses. The A1 and V1 regions are shown averaged across hemispheres. The results from the non-represented dimensions within each region (orientation and finger in A1, tone and finger in V1, orientation and tone in M1) have also been averaged. One sample *t*-tests comparing classification accuracy in each condition to chance (50%) showed that in each region, accuracy was significantly above chance for the represented dimensions, but not for non-represented dimensions, suggesting that the analysis was both sensitive and specific despite the relatively low number of events. This was only true for the data collected using the gradient echo (GE) sequences however, and classification accuracy was at chance levels in the data collected using the spin echo (SE) sequences.

Classification accuracy in each region was analysed further using repeated measures ANOVAs. In the A1 regions, the analysis was carried out using four factors: sequence type (GE vs. SE), resolution (standard vs. high), hemisphere (left vs. right), and contrast (orientation, tone, finger). The results showed significant main effects of sequence type (GE > SE; *F*(1,10) = 25.15, *p* < 0.001) and contrast (*F*(2,20) = 19.41, *p* < 0.001). A significant interaction was also observed between sequence type and contrast (*F*(2,20) = 16.61, *p* < 0.001). Paired sample *t*-tests showed that accuracy of tone classification in GE sequences was greater than accuracy in all other conditions (*p* < 0.001 in all cases) and that there were no differences in accuracy amongst the remaining conditions (*p* > 0.25 in all cases). Finally, a significant interaction was also observed between resolution and contrast (*F*(2,20) = 4.33, *p* < 0.05). While tones were classified significantly more accurately than either orientations or fingers at both resolutions (paired *t*-tests, all *p* < 0.05), the size of the difference was larger in the standard resolution sequences (tone = 57.6%, other = 48.8%) than high resolution sequences (tone = 56.0%, other = 50.8%).

A similar analysis carried out on the classification results from V1 also showed significant main effects of sequence type (GE > SE; *F*(1,10) = 16.87, *p* < 0.01) and contrast (*F*(2,20) = 5.38, *p* < 0.05), together with a significant interaction between sequence and contrast (*F*(2,20) = 9.72, *p* < 0.001). Paired *t*-tests showed that classification of orientation in the GE sequences was significantly more accurate than classification in any other condition (*p* < 0.05 in all cases).

Analysis of data from M1 was carried out using a two-way repeated measures ANOVA with sequence type (GE standard resolution, GE high resolution, SE standard resolution) and contrast (orientation, tone, finger) as factors. A significant main effect of contrast (*F*(2,20) = 8.12, *p* < 0.01) and a significant interaction between sequence and contrast (*F*(4,40) = 3.98, *p* < 0.01) were observed. Paired *t*-tests showed that classification of finger was significantly more accurate than classification of both tone and orientation in standard resolution GE data (both *p* < 0.05) and more accurate than classification of orientation in the high resolution GE data (*p* < 0.05). No significant differences were observed amongst any of the contrast types in the SE data (all *p* > 0.5). In addition, classification of finger was significantly more accurate in both sets of GE data than in the SE data (both *p* < 0.05), while there was no difference between classification accuracy in the 2 sets of GE data (*p* > 0.45).

A series of univariate analyses were also carried out in order to examine whether the pattern classification results were driven by global differences in activity across each region. For each region, the general linear model described above (see “Data analysis”) was applied to the mean time course across all voxels. The parameter estimates obtained from this model were then entered into a series of contrasts comparing the values of orientation, tone and finger. Contrast values from each participant (averaged across hemispheres in the A1 and V1 regions) were entered into group analyses and assessed using one sample *t*-tests. The only significant differences were finger in M1 during the high resolution GE sequence (middle > index, *p* < 0.05), and orientation in A1 during the high resolution SE sequence (horizontal > vertical, *p* < 0.05). Repeating the analyses described above using the univariate contrast values instead of classification accuracy scores revealed no significant effects. Correlations were also carried out to examine the relationship between absolute univariate contrast values (representing the size of any global bias towards either stimulus value) and classification accuracy scores. The only significant relationship between the two sets of scores occurred for tones in V1 during the standard resolution GE sequence (*p* < 0.05).

### Spatial smoothing and pattern classification

**Fig. 3:**
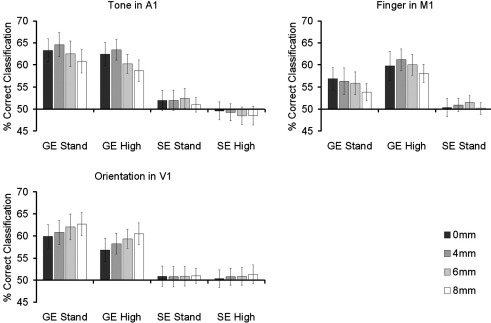
Mean classification accuracy rates in unsmoothed data and at three different levels of spatial smoothing. Smoothing was carried out using Gaussian kernels of either 4, 6 or 8 mm FWHM. The effect of smoothing varied across regions, producing a decrease in classification accuracy in A1 and M1, but an increase in accuracy in V1. Error bars represent 1 standard error of the mean (calculated across participants).

Following the results of Op de Beeck et al. (2008), Op de Beeck (2010) and Gardner et al. (2006), an analysis was also carried out to investigate the effects of spatial smoothing on classification accuracy. The realigned, slice timed data from each participant were convolved with Gaussian kernels of three different widths (4, 6, and 8 mm FWHM), and the classification analyses described in the Data Analysis section were repeated using this smoothed data. Fig. 3 shows the results for the represented dimension in each region at each kernel width (results in A1 and V1 have been averaged over hemispheres).

The results shown in Fig. 3 suggest that spatial smoothing had an impact on classification accuracy that varied between regions. In order to investigate this further, the data from the GE sequences were analysed using a series of repeated measures ANOVAs. Tone classification accuracy in A1 was modelled using three factors: spatial resolution (standard vs. high), hemisphere (left vs. right), and FWHM (0, 4, 6, and 8 mm). The only significant finding was a main effect of FWHM (*F*(3,30) = 13.68, *p* < 0.001). As Fig. 3 shows, this effect did not reflect a simple monotonic trend. While there was a slight (but non-significant, paired *t*-test *p* > 0.2) improvement in performance going from no smoothing to a 4 mm kernel, this was followed by a decrease in accuracy when the kernel width was increased to 6 mm (4 mm vs. 6 mm, paired *t*-test *p* < 0.001), followed by a further decrease when the width was increased again to 8 mm (6 mm vs. 8 mm, paired *t*-test *p* < 0.01). The accuracy of finger classification in M1 showed a weaker version of the same pattern. A two-way repeated measures ANOVA (spatial resolution × FWHM) showed a main effect of FWHM (*F*(3,30) = 3.29, *p* < 0.05), but post hoc *t*-tests showed that the decline in classifier performance was only significant between smoothing at 4 mm and smoothing at 8 mm (*p* < 0.05), and between smoothing at 6 mm and smoothing at 8 mm (*p* < 0.05).

In contrast to the pattern observed in A1 and M1, increasing the width of the smoothing kernel appeared to produce a consistent improvement in classification accuracy of orientation in V1. A three-way repeated measures ANOVA (spatial resolution × hemisphere × FWHM) found a significant main effect of FWHM (*F*(3,30) = 4.74, *p* < 0.01) and also a significant linear contrast across all levels of FWHM (*F*(1,10) = 5.26, *p* < *p* < 0.05).

Repeating these analyses using data from the spin echo sequences produced no significant results in any region.

### Time course of haemodynamic response in gradient and spin echo

Recent results suggest the shape of the haemodynamic response may differ in gradient and spin echo data (Hulvershorn et al., 2005). As the classifications described above were based on parameter estimates obtained using the SPM5 canonical haemodynamic response function (HRF), this raises the possibility that the poorer classification accuracies observed for the SE data were a result of the canonical HRF providing a poorer fit to the SE haemodynamic response. In order to investigate this issue, event related time courses were derived for each sequence type in each of the regions of interest. These were obtained by taking the mean time course across all voxels within each of the regions. The evoked haemodynamic response for each experimental trial was then obtained by sampling the mean time course of each region at 20 1-s bins time locked to the onset of each trial. For each participant, the evoked response for each region was found by taking the mean event related response at each time point and scaling by the standard error at each time point. Finally, the value in the first bin was subtracted from the value at each of the other bins.

**Fig. 4:**
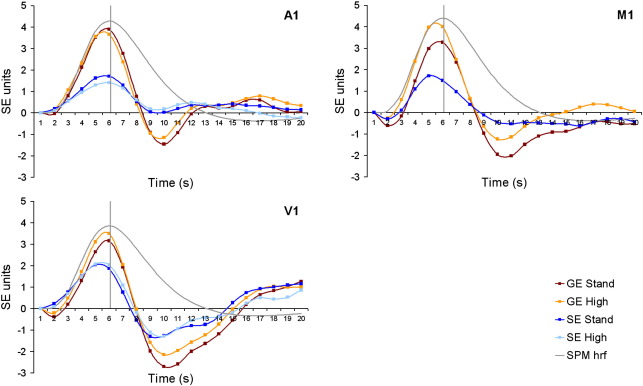
Event related time courses for each of the four sequence types plotted alongside the haemodynamic response function (HRF) obtained using the default parameters in SPM5. In all cases there was a more rapid decline from peak response in the empirically derived response functions than in the canonical HRF. In addition, the spin echo data showed a shorter peak latency in V1 and M1. For clarity of display, the canonical HRF function has been arbitrarily scaled to have a peak response 10% greater than the maximum height of the empirically derived event related time courses.

**Fig. 5:**
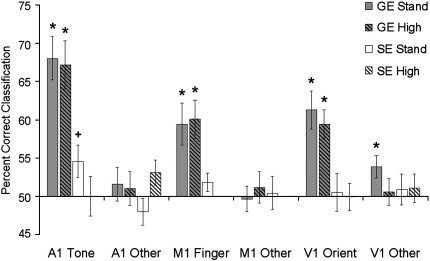
Mean classification accuracy results obtained using basis functions modelled on the empirically derived event related time courses shown in Fig. 4. In A1, this approach produced an increase in mean accuracy of 4.72% in the standard resolution GE data, 4.75% in the high resolution GE data, 2.67% in the standard resolution SE data, and 0.49% in the high resolution SE data. The corresponding figures in M1 were 2.61% (standard GE), 0.3% (high GE), and 1.52% (standard SE). In V1, there were increases in the GE data (standard resolution = 1.36%, high resolution = 2.62%), but slight decreases in the SE data (standard resolution = −0.34%, high resolution = −0.35%). * = 1 sample *t*-test against chance (50%), *p* < 0.05, ^+^*p* = 0.054. Error bars represent 1 standard error of the mean (calculated across participants).

Fig. 4 shows the resulting event related time courses alongside the canonical HRF used by SPM5. The peak of the evoked haemodynamic responses appeared to correspond with the peak of the canonical HRF most closely for the GE data in A1 and V1, while the peak evoked response in the SE data occurred earlier than the peak of the canonical HRF, particularly in M1 and V1. In order to take these differences into account, the classification analyses described in the Data Analysis section were carried out again using basis functions that were more closely matched to the event related time courses for each sequence type (this method was chosen in preference to the more flexible approach of using an FIR basis set in order to control the total number of columns in the design matrix). This analysis led to an improvement in classification performance of up to 4.8% (Fig. 5) and produced a marginally significant above chance discrimination of tone in A1 for the standard resolution SE data (*t*(10) = 2.18, *p* = 0.054). Accuracies were still significantly higher in the GE data however, and repeating the ANOVAs described above with the new accuracy ratings produced similar main effects of sequence and contrast, as well as significant interactions between sequence and contrast.

## Discussion

The current report describes the effect of several factors on multivariate pattern classification in primary sensory and motor areas. One of the most salient results was the significant difference between classification accuracies obtained from data collected using a gradient echo sequence and those obtained from data collected using a spin echo sequence. While it was possible to detect differences between line orientation in V1, tone frequency in A1 and finger in M1 using the gradient echo sequence, classification accuracies were not significantly different from chance in the spin echo data. It is possible that improvements in the signal-to-noise ratio (for example, by using surface coils rather than a birdcage head coil), or contrast-to-noise ratio (for example, by including more observations) might allow above chance classification in the spin echo data, although as any increase in the signal- or contrast-to-noise ratios would also be beneficial for the gradient echo data, the difference in accuracy between the two sequence types should remain.

From a methodological perspective, the current results suggest that the gradient echo sequence may be preferable for multivariate pattern analyses, at least at the field strength and spatial resolution currently used for the majority of fMRI studies. The results also suggest that the signal used in pattern analyses contains a considerable contribution from larger draining veins, since classifier performance showed a marked decline when this signal was reduced in the spin echo data. In this respect the result is consistent with the findings reported by Shmuel et al. (2010) examining the spatial distribution of information about ocular dominance in V1. By calculating the discriminative power of individual voxels and comparing the results with the probability that those voxels belonged to regions containing macroscopic blood vessels, the authors demonstrated that peaks of discriminative power were present in voxels containing larger draining veins.

While the extra-vascular signal around larger draining veins should have been eliminated in the spin echo data, previous work suggests that with parameters similar to those used in the current study, there would still be some signal originating from inside larger veins (Norris et al., 2002; Duong et al., 2003; Jochimsen et al., 2004). This intravascular signal is expected to be weaker than the extra-vascular signal however, and the current results suggest that it was not strong enough on its own to provide a useful source of information. Further work using acquisition sequences that suppressed intravascular contributions (for example comparing gradient echo data with and without diffusion gradients) would be necessary to determine the relative contributions of intra- and extra-vascular signals, in particular whether either source in isolation could provide sufficient signal for reliable pattern classification. Similarly, comparisons of gradient and spin echo data in the presence of diffusion gradients (or at high magnetic field strengths) would be necessary to determine the effect of completely eliminating any signal from larger vessels.

While the current results demonstrate the importance of the vascular signal at 3T, they do not rule out the possibility of gray matter or microvascular contributions to pattern analysis. In the study described above, Shmuel et al. (2010) found that discriminative power was also present in voxels that contained mainly gray matter, and it is possible that multiple sources of information exist. Importantly, Shmuel et al. (2010) carried out their study at 7T, and it is likely that the relative contributions of gray matter and draining veins will depend on magnetic field strength (Duong et al., 2003). The current results suggest that at 3T pattern information is provided mainly by signals from larger draining veins. At higher field strengths, however, it is possible that the relative increase in the strength of the microvascular signal (Lee et al., 1999; Duong et al., 2003; Jochimsen et al., 2004) may produce a situation that more closely resembles the biased sampling model, with patterns of spatially localised signals originating from different populations of cells. In line with this idea, another study carried out by Shmuel et al. (2007b) at 7T reported equally high classification accuracy for ocular dominance columns in both spin and gradient echo data.

In addition to the effect of sequence type, the study also investigated the role of spatial smoothing on classification accuracy, and the results present a complex picture, with the precise effects of smoothing depending on the region and stimulus dimension in question. The results from V1 are consistent with those recently reported by Op de Beeck (2010) and Gardner et al. (2006) in demonstrating that spatial smoothing does not harm classifier performance in V1. Indeed, in the current study, classification of orientation in V1 actually appeared to be enhanced by spatial smoothing. This effect was specific to V1 however, and increasing the size of the smoothing kernel reduced the accuracy of tone classification in A1 and finger classification in M1.

On first inspection, the different effects of smoothing appear somewhat surprising given what is known about the functional organisation of the three regions. In human primary auditory cortex, functional imaging has revealed mirror symmetric tonotopic maps where particular frequency bands are represented by populations centred on locations separated by several mm (Formisano et al., 2003). The topography of M1 appears to be organised on a similar spatial scale, with individual fingers represented by partially overlapping populations centred on foci that are approximately 2–4 mm apart (Indovina and Sanes, 2001; Dechent and Frahm, 2003). In contrast, representation of orientation in V1 appears to be organised into much smaller functional units (∼ 750 µm in width) that are widely distributed over an area of several square cm (Yacoub et al., 2008).

Assuming that pattern analysis measures differences in the distribution of these functional units, the matched filter theorem would predict that V1 should be more vulnerable to smoothing than that in either A1 or M1, contrary to the observed pattern of results. Crucially, representation in V1 is periodic, compared to the relatively localised representations in A1 and M1 where individual tones or fingers are maximally represented at distinct spatial locations. The repeating pattern in V1 raises the possibility of either a pre-existing low frequency component to the information (Op de Beeck, 2010), or that high frequency information was aliased into low frequency components (Kriegeskorte et al., 2010). In either case, smoothing could have produced a relative amplification of low frequency information in V1, while diluting the more localised information present in A1 and M1. Interestingly, there was some evidence that both A1 and M1 showed an initial increase in accuracy when a small amount of smoothing (4 mm FWHM Gaussian kernel) was introduced. Given the predictions of the matched filter theorem, it would be interesting to test whether the optimum level of smoothing in these regions depends on the particular tones or fingers that are compared, in particular whether comparisons between tones or fingers that are represented by populations with greater spatial separation benefit from more smoothing.

Another possibility for the pattern of results in V1 is that there was a greater representation of one particular orientation, producing global differences in signal between the two conditions. Previous results suggest that there may be differences in the representation of horizontal and vertical orientations, although the picture is inconsistent, with some studies reporting an over-representation of vertical orientations (Yacoub et al., 2008), some an over-representation of horizontal orientations (Serences et al., 2009), and others reporting similar levels of signal change for horizontal and vertical orientations (Furmanski and Engel, 2000). In addition, the univariate analyses carried out in the current study showed that there were no global differences in activation between the two orientations.

The current study focuses on primary sensory and motor areas, and the relative contribution of micro- and macro-vascular signals in regions of the brain with less differentiated neural topographies remains an open question. Together with several other recent reports however, the current findings highlight the importance of taking both into account as potential sources of information. One relatively low cost strategy for future studies focussing on areas where the underlying neural topography, or patterns of neuro-vascular coupling, are unclear could be to use a dual echo sequence with near simultaneous acquisition of both gradient and spin echo data in each TR (Bandettini et al., 1993). At high field strengths this could provide a direct estimate of the vascular contribution to pattern separation. This type of approach could also be used to give an estimate of the mean vessel size within each voxel (e.g., Jochimsen and Moller, 2008), and a comparison of classification results from voxels containing different vessel sizes could provide further important insights into the source of pattern separation.

## Figures and Tables

**Table 1:** Behavioural results showing the percentage of responses made, the percentage of correct responses, and average reaction time in each of the eight stimulus conditions. Results have been averaged across all four scanning sequences. SE = standard error of the mean, calculated across subjects.

Mapping	Orient	Tone	% Made		% Correct		Reaction time (ms)	
Average	SE	Average	SE	Average	SE
1	Vertical	Low	97.6	0.8	92.4	2.5	1068	57
High	98.8	0.5	93.7	3.3	870	54
Horizontal	Low	96.8	0.9	90.7	2.7	1136	46
High	97.9	0.8	92.6	2.9	1075	48
2	Vertical	Low	96.2	0.9	86.8	3.7	1165	44
High	97.6	0.7	91.7	3.3	1054	42
Horizontal	Low	96	0.9	90.3	3.1	1112	54
High	97.6	0.8	91.6	2.4	976	54
